# Estimation and Comparison of Current and Future Racial/Ethnic Representation in the US Health Care Workforce

**DOI:** 10.1001/jamanetworkopen.2021.3789

**Published:** 2021-03-31

**Authors:** Edward Salsberg, Chelsea Richwine, Sara Westergaard, Maria Portela Martinez, Toyese Oyeyemi, Anushree Vichare, Candice P. Chen

**Affiliations:** 1 Fitzhugh Mullan Institute for Health Workforce Equity, The George Washington University Milken Institute School of Public Health, Washington, DC; 2 Department of Emergency Medicine, The George Washington University School of Medicine and Health Sciences, Washington, DC; 3 Department of Health Policy and Management, The George Washington University Milken Institute School of Public Health, Washington, DC

## Abstract

**Importance:**

The COVID-19 pandemic coupled with health disparities have highlighted the disproportionate burden of disease among Black, Hispanic, and Native American (ie, American Indian or Alaska Native) populations. Increasing transparency around the representation of these populations in health care professions may encourage efforts to increase diversity that could improve cultural competence among health care professionals and reduce health disparities.

**Objective:**

To estimate the racial/ethnic diversity of the current health care workforce and the graduate pipeline for 10 health care professions and to evaluate whether the diversity of the pipeline suggests greater representation of Black, Hispanic, and Native American populations in the future.

**Design, Setting, and Participants:**

This cross-sectional study used weighted data from the 2019 American Community Survey (ACS) to compare the diversity of 10 health care occupations (advanced practice registered nurses, dentists, occupational therapists, pharmacists, physical therapists, physician assistants, physicians, registered nurses, respiratory therapists, and speech-language pathologists) with the diversity of the US working-age population, and 2019 data from the Integrated Postsecondary Education Data System (IPEDS) were used to compare the diversity of graduates with that of the US population of graduation age. Data from the IPEDS included all awards and degrees conferred between July 1, 2018, and June 30, 2019, in the US.

**Main Outcomes and Measures:**

A health workforce diversity index (diversity index) was developed to compare the racial/ethnic diversity of the 10 health care professions (or the graduates in the pipeline) analyzed with the racial/ethnic diversity of the current working-age population (or average student-age population). For the current workforce, the index was the ratio of current workers in a health occupation to the total working-age population by racial/ethnic group. For new graduates, the index was the ratio of recent graduates to the population aged 20 to 35 years by racial/ethnic group. A value equal to 1 indicated equal representation of the racial/ethnic groups in the current workforce (or pipeline) compared with the working-age population.

**Results:**

The study sample obtained from the 2019 ACS comprised a weighted total count of 148 358 252 individuals aged 20 to 65 years (White individuals: 89 756 689; Black individuals: 17 916 227; Hispanic individuals: 26 953 648; and Native American individuals: 1 108 404) who were working or searching for work and a weighted total count of 71 608 009 individuals aged 20 to 35 years (White individuals: 38 995 242; Black individuals: 9 830 765; Hispanic individuals: 15 257 274; and Native American individuals: 650 221) in the educational pipeline. Among the 10 professions assessed, the mean diversity index for Black people was 0.54 in the current workforce and in the educational pipeline. In 5 of 10 health care professions, representation of Black graduates was lower than representation in the current workforce (eg, occupational therapy: 0.31 vs 0.50). The mean diversity index for Hispanic people was 0.34 in the current workforce; it improved to 0.48 in the educational pipeline but remained lower than 0.50 in 6 of 10 professions, including physical therapy (0.33). The mean diversity index for Native American people was 0.54 in the current workforce and increased to 0.57 in the educational pipeline.

**Conclusions and Relevance:**

This study found that Black, Hispanic, and Native American people were underrepresented in the 10 health care professions analyzed. Although some professions had greater diversity than others and there appeared to be improvement among graduates in the educational pipeline compared with the current workforce, additional policies are needed to further strengthen and support a workforce that is more representative of the population.

## Key Points

**Question:**

Are Black, Hispanic, and Native American (ie, American Indian or Alaska Native) populations underrepresented in the health care professions in the US, and does the educational pipeline show greater representation of these groups in the future health care workforce?

**Findings:**

In this cross-sectional study of 2019 data from the American Community Survey and the Integrated Postsecondary Education Data System, Black, Hispanic, and Native American people were underrepresented in the 10 health care professions analyzed. Although the educational pipeline shows some limited improvement, underrepresentation of these groups persists.

**Meaning:**

Results of this study suggest that additional efforts are needed to increase the representation of Black, Hispanic, and Native American people in the health care professions; measuring and reporting on representation of these groups in the health care workforce and educational pipeline may encourage these efforts.

## Teaser

This cross-sectional study used 2019 data from the American Community Survey and the Integrated Postsecondary Education Data System to estimate and compare the representation of Black, Hispanic, and Native American minority groups in 10 health care professions and in the educational pipeline for these professions.

## Introduction

Recent reports describing the health disparities faced by racial/ethnic minority groups during the COVID-19 pandemic has added to decades of literature demonstrating differential access to health care services and health outcomes by race and ethnicity.^1,2,3,4,5,6,7^ This unequal burden extends to health care workers; studies have revealed that Black, Hispanic, and Native American (ie, American Indian or Alaska Native) frontline health care professionals have been disproportionately affected by COVID-19.^8,9^

A substantial body of literature suggests that fostering a diverse and inclusive workforce is critical to increasing access to care and improving aspects of health care quality among underserved populations.^10,11,12,13^ Studies have demonstrated that physicians and dentists from underrepresented minority groups are more likely to practice in high-need specialties and in underserved communities.^14,15^ Student body diversity has been associated with better overall student preparation to care for minority populations and an endorsement of equitable access to care.^16^ Some studies have suggested that a diverse workforce may improve health care professionals’ cultural competence and better prepare them to respond to the needs of the entire population.^17^ Literature on patient-physician concordance suggests that diversity may be important for quality of care with regard to patient communication, preventive care, and patient satisfaction.^18,19^ A diverse workforce with a broader set of experiences in leadership roles can also aid in shaping research and policy agendas.^20^

Although studies have informed policies to improve diversity, most focus on the medical workforce, with fewer studies examining the racial/ ethnic diversity of other health care professions.^19,21,22,23^ Furthermore, few studies have systematically assessed the diversity of multiple professions or compared performance over time.

By examining the racial/ethnic diversity of the current workforce, this analysis explores the representation of Black, Hispanic, and Native American people in the current health care workforce. Although other racial/ethnic populations are of interest and play important roles in the health care system, this analysis focuses on population groups (Black, Hispanic, and Native American) that have been historically identified as underrepresented in health care professions that require a higher level of educational attainment. This analysis also estimates the representation of these minority groups among recent graduates of health care professional programs (ie, the educational pipeline into the health care professions) to ascertain whether these graduates may change representation of Black, Hispanic, and Native American populations in the future workforce.

## Methods

### Data Sources

In this cross-sectional study, publicly available data from the 2019 American Community Survey (ACS) and the 2019 Integrated Postsecondary Education Data System (IPEDS) were used to estimate the current racial/ethnic profile of 10 health care occupations: advanced practice registered nurses, dentists, occupational therapists (OTs), pharmacists, physical therapists (PTs), physician assistants (PAs), physicians, registered nurses, respiratory therapists (RTs), speech-language pathologists.^24,25^ The ACS is the US Census Bureau’s survey of all individuals in the US; it samples about 1% of the population annually. Population data are weighted by the Census Bureau to make the information representative of the nation. Race/ethnicity were self-reported. The IPEDS is operated by the National Center for Education Statistics within the US Department of Education. Data, including the race/ethnicity of graduates, are collected annually from nearly all postsecondary educational programs. Data from the IPEDS included all awards and degrees conferred between July 1, 2018 and June 30, 2019 in the US. We compared programs reporting to the IPEDS with accredited programs in selected health care professions and found that data were available in the IPEDS on all accredited programs. The programs collected data on the race/ethnicity of graduates according to degree type and major or area of study. Because this study used publicly available data sets, the institutional review board of The George Washington University did not not consider this study to be human participant research, and the requirements for review and informed consent were waived. This study follows the Strengthening the Reporting of Observational Studies in Epidemiology (STROBE) reporting guideline.^26^

Both the ACS and IPEDS use separate questions to collect data on race and ethnicity. For race, both databases list the following categories as options: American Indian/Alaska Native, Asian, Black or African American, Native Hawaiian/Pacific Islander, and White. For this analysis, we use the categories of Black or African American (referred to as Black in this article) and American Indian/Alaska Native (referred to as Native American in this article). To ascertain ethnicity, the ACS asks if the individual is of Hispanic, Latino, or Spanish ethnicity, whereas the IPEDS asks if the individual is of Hispanic ethnicity. Herein, we refer to individuals in both data sets as Hispanic. The IPEDS also collects data and reports on the number of non–resident individuals, which is not a category in the ACS; therefore, we excluded these individuals from this analysis. We also excluded individuals who self-reported race as mixed, other race, or multiple races.

This analysis focused on the 10 largest health care professions defined by the US Bureau of Labor Statistics’ Standard Occupational Classification as being health diagnosing and treating practitioners who require a postsecondary degree.^27^ To estimate the racial/ethnic diversity of the current health care workforce, we used data from the 2019 ACS. To estimate the diversity of the educational pipeline, we created a crosswalk of likely degrees that lead to the previously mentioned professions and used the 2019 IPEDS data to determine the number of graduates with those degrees.

### Development of a Health Workforce Diversity Index

To quantify the extent of racial/ethnic representation in the health care workforce compared with the general population, we developed a health workforce diversity index (hereafter referred to as diversity index). For the current health care workforce, this index was calculated as the ratio of current workers in a health occupation to the total working-age population (ie, individuals aged 20-65 years who were working or searching for work) by racial/ethnic group. The diversity index for new graduates was calculated as the ratio of recent graduates to the total population (aged 20-35 years) by racial/ethnic group. Although the typical age of graduation varies by profession, most graduates fall within the age range of 20 to 35 years. Some professions, such as respiratory therapy and nursing, require an associate degree, and some students begin after graduating from high school. Other professions, such as medicine and dentistry, require a postgraduate education, and many graduates are in their early 30s. For example, 12.1% of the current US working-age population is composed of Black individuals (95% CI, 11.99-12.16); therefore, if 12.1% of practitioners in a health care profession are Black, the diversity index would be equal to 1. If 6% of practitioners are Black, the diversity index would be equal to 0.5. Hispanic people comprise 18.2% of the working-age population (95% CI, 18.08-18.26). If 12.1% of practitioners in a health care profession are Hispanic, the diversity index would be 0.66, and if 6.0% of practitioners in a health care profession are Hispanic, the diversity index would be 0.33.

Occasionally, we noted the number of professions with a diversity index lower than 0.5 or lower than 0.33; this is an a priori benchmark showing how far the current diversity level is from parity (ie, diversity of the health care workforce would be equal to the diversity of the population). New graduates reflect the inflow of individuals into a profession and are referred to as the educational pipeline. We compared the diversity index of recent graduates of health care professional programs with the diversity index of the current workforce to ascertain whether the future health care workforce is likely to be more or less diverse than the current health care workforce. The use of an index to measure the extent of diversity with parity equal to 1 was recently described in a study of faculty diversity at academic medical centers.^28^

### Statistical Analysis

We calculated SEs and 95% CIs for the population estimates from the ACS using Stata, version 16 (StataCorp LLC). We assumed that the sample was representative of the population if the SEs were small (eg, <1). The analysis of the recent graduates is based on the number of graduates with degrees in each of the specific occupations during the reporting year (2019) who were included in the IPEDS. Because the IPEDS collects data on all graduates, no statistical tests were performed on this data.

## Results

As mentioned previously, population data from the 2019 ACS were weighted. The study sample comprised 148 358 252 individuals aged 20 to 65 years (White individuals: 89 756 689; Black individuals: 17 916 227; Hispanic individuals: 26 953 648; and Native American individuals: 1 108 404) who were working or searching for work and 71 608 009 individuals aged 20 to 35 years (White individuals: 38 995 242; Black individuals: 9 830 765; Hispanic individuals: 15 257 274; and Native American individuals: 650 221) in the educational pipeline. In 2019, approximately 12.1% (95% CI, 11.99-12.16) of the US working-age population was composed of Black individuals (Table 1). In contrast, among the 10 occupations reviewed, representation of Black individuals ranged from 3.3% (95% CI, 2.50-4.41) for PTs to 11.4% (95% CI, 8.81 to 14.66) for RTs (Table 2 and Figure, A). The mean diversity index was 0.54 for Black health workers for the 10 professions. Hispanic individuals accounted for 18.2% (95% CI, 18.08-18.26) of the working-age population (Table 1); however, Hispanic representation among the 10 occupations we reviewed ranged from 3.4% for PTs (95% CI, 2.62-4.27) to 10.8% for RTs (95% CI, 8.45-13.73) (Table 2, Figure, B). The mean diversity index was 0.34 for Hispanic health workers for the 10 professions. Native American individuals accounted for 0.6% of the working-age population in 2019 (95% CI, 0.56-0.59) (Table 1), whereas Native American representation among the 10 occupations analyzed ranged from 0% for PTs (95% CI, 0-0.16) to 0.9% for RTs (95% CI, 0.29-2.98), resulting in a mean diversity index of 0.54 (Table 2).

**Table 1.** Workforce and Educational Pipeline Population Estimates Based on 2019 American Community Survey (ACS) Data^a^

Race/ethnicity	Workforce		Educational pipeline	
	Weighted count	Weighted % (SE) [95% CI]^b^	Weighted count	Weighted % (SE) [95% CI]^b^
White	89 756 689	60.5 (0.06) [60.39-60.61]	38 995 242	54.5 (0.09) [54.29-54.63]
Black	17 916 227	12.1 (0.04) [11.99-12.16]	9 830 765	13.7 (0.07) [13.60-13.86]
Asian	9 319 708	6.3 (0.03) [6.23-6.33]	4 642 874	6.5 (0.04) [6.40-6.56]
AIAN	850 074	0.6 (0.01) [0.56-0.59]	505 207	0.7 (0.01) [0.68-0.73]
NHPI	258 330	0.2 (0.01) [0.16-0.18]	145 014	0.2 (0.01) [0.19-0.22]
Multiple race/other	3 303 576	2.2 (0.02) [2.19-2.26]	2 231 633	3.1 (0.03) [3.06-3.18]
Hispanic	26 953 648	18.2 (0.05) [18.08-18.26]	15 257 274	21.3 (0.07) [21.16-21.45]
Total	148 358 252	100	71 608 009	100

Abbreviations: AIAN, American Indian or Alaska Native; NHPI, Native Hawaiian or Pacific Islander.

^a^ All estimates came from the 2019 one-year ACS. General workforce estimates included individuals aged 20 to 65 years who were working or searching for work. Total educational pipeline estimates included individuals aged 20 to 35 years (the potential student population).

^b^ The SE is the linearized SE of the column percentage, and the 95% CI represents the lower and upper 95% confidence bound for the column percentage.

**Table 2.** Workforce Estimates of Health Diagnosing and Treating Practitioners Based on 2019 American Community Survey Data

Practitioner	Race/ethnicity, % (SE) [95% CI]			
	White	Black	Native American	Hispanic
Advanced practice registered nurse	79.4 (1.10) [77.15-81.47]	7.3 (0.87) [5.80-9.22]	0.3 (0.16) [0.12-0.84]	5.5 (0.58) [4.45-6.74]
Dentist	68.7 (1.52) [65.60-71.56]	4.4 (0.88) [2.95-6.49]	0.1 (0.05) [0.01-0.29]	5.7 (0.72) [4.43-7.28]
Pharmacist	65.4 (1.09) [63.22-67.51]	7.5 (0.72) [6.23-9.07]	0.2 (0.08) [0.07-0.45]	3.7 (0.41) [2.99-4.63]
Physician	62.4 (0.65) [61.06-63.63]	5.2 (0.37) [4.50-5.96]	0.1 (0.05) [0.047-.26]	6.9 (0.35) [6.27-7.65]
Physician assistant	75.9 (1.46) [72.97-78.68]	4.5 (0.82) [3.11-6.39]	0.5 (0.24) [0.23-1.29]	7.3 (0.87) [5.77-9.21]
Occupational therapist	80.5 (1.42) [77.60-83.16]	6.1 (1.03) [4.35-8.45]	0.2 (0.17) [0.02-1.19]	5.2 (0.78) [3.90-7.0]
Physical therapist	76.7 (1.06) [74.54-78.71]	3.3 (0.48) [2.50-4.41]	0 (0.02) [0-0.16]	3.3 (0.42) [2.62-4.27]
Respiratory therapist	66.3 (2.03) [62.19-70.16]	11.4 (1.48) [8.81-14.66]	0.9 (0.56) [0.29-2.98]	10.8 (1.34) [8.45-13.73]
Speech-language pathologist	84.4 (1.15) [82.00-86.52]	4.7 (0.78) [3.37-6.47]	0.5 (0.28) [0.20-1.48]	6.4 (0.74) [5.10-8.03]
Registered nurse	68.9 (0.38) [68.17-69.64]	11.3 (0.29) [10.75-11.91]	0.4 (0.05) [0.29-0.47]	7.8 (0.22) [7.33-8.21]

**Figure.**
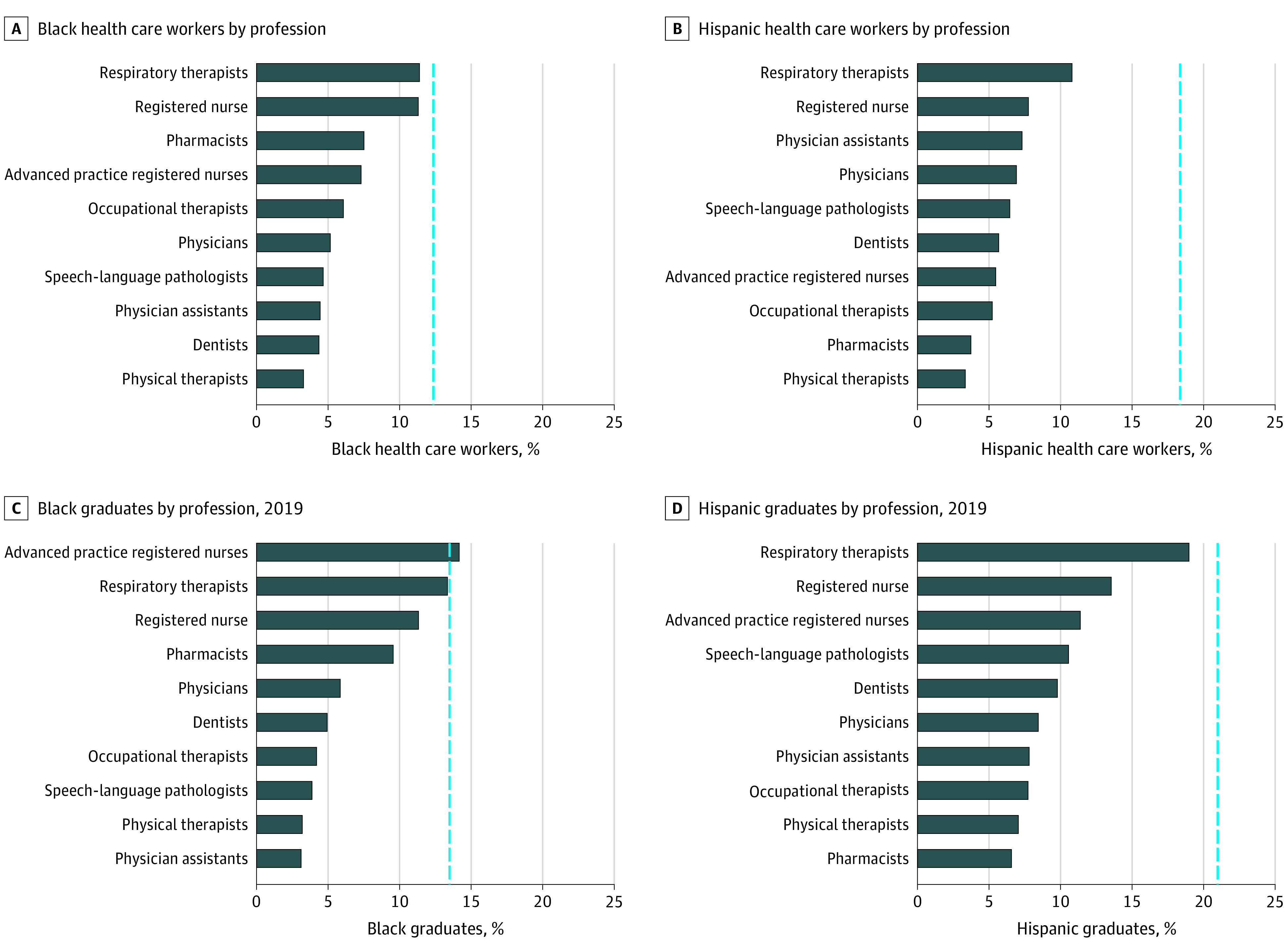
Representation of Black and Hispanic Individuals in the Health Care Workforce and Health Care Profession Graduates in 2019 A and B, For the current workforce, the vertical line represents the percentage of the working-age population who are Black or Hispanic individuals. In 2019, Black individuals comprised 12.1% and Hispanic individuals comprised 18.2% of the US health care workforce. C and D, For the educational pipeline of 2019 graduates, the vertical line represents the percentage of the general population between the ages of 20 and 35 years who are Black or Hispanic individuals. In 2019, 13.7% of the US population aged 20 to 35 years was Black (non-Hispanic), and 21.3% of the population was Hispanic.

The diversity index for the current health care workforce was lower than 0.50 in 9 of 10 professions (all except respiratory therapy) for Hispanic health workers. For 5 occupations (advanced practice registered nurses, dentists, pharmacists, OTs, and PTs), Hispanic representation was lower than one-third of their representation in the working-age population (Table 3). For Black and Native American health workers, the diversity index for the current health care workforce was lower than 0.50 in 5 of 10 occupations (dentist, pharmacist, physician, OT, and PT).

**Table 3.** Health Workforce Diversity Index for the 2019 Health Care Workforce and Educational Pipeline

Occupation	White			Black			Hispanic			Native American		
	Workforce^a^	Pipeline^b^	Change^c^	Workforce^a^	Pipeline^b^	Change^c^	Workforce^a^	Pipeline^b^	Change^c^	Workforce^a^	Pipeline^b^	Change^c^
Advanced practice registered nurse	1.31	1.17	−0.14	0.61	1.03	0.42	0.30	0.53	0.23	0.56	0.70	0.14
Dentist	1.13	1.04	−0.10	0.36	0.36	0.00	0.31	0.46	0.15	0.09	0.40	0.30
Pharmacist	1.08	0.98	−0.10	0.62	0.70	0.07	0.20	0.31	0.10	0.31	0.53	0.21
Physician	1.03	1.09	0.06	0.43	0.43	0.00	0.38	0.40	0.02	0.19	0.51	0.32
Physician assistant	1.26	1.42	0.16	0.37	0.23	−0.14	0.40	0.37	−0.03	0.94	0.48	−0.47
Occupational therapist	1.33	1.44	0.10	0.50	0.31	−0.20	0.29	0.36	0.07	0.30	0.24	−0.06
Physical therapist	1.27	1.40	0.13	0.28	0.23	−0.04	0.18	0.33	0.15	0.04	0.36	0.32
Respiratory therapist	1.10	1.00	−0.10	0.94	0.97	0.03	0.60	0.89	0.30	1.64	1.05	−0.59
Speech-language pathologist	1.39	1.45	0.06	0.39	0.28	−0.11	0.35	0.50	0.14	0.96	0.54	−0.42
Registered nurse	1.14	1.20	0.06	0.94	0.82	−0.11	0.43	0.64	0.21	0.65	0.81	0.17

^a^ For each occupation, the diversity index for the workforce is the percentage of practitioners in a given racial/ethnic group (using 2019 American Community Survey data) ÷ that group’s representation in the general workforce population aged 20 to 65 years (eg, the percentage of Black advanced practice registered nurses ÷ the percentage of Black individuals in the general workforce).

^b^ For each occupation category, the diversity index for the educational pipeline is the percentage of recent graduates in a given racial/ethnic group (using 2019 Integrated Postsecondary Education Data System data) ÷ that group’s representation in the general population aged 20 to 35 years (eg, the percentage of Asian physician assistant graduates ÷ the percentage of Asian individuals in the population aged 20 to 35 years).

^c^ The change indicates the increase or decrease in the diversity index for the pipeline (compared with the workforce) for each occupation.

In 2019, approximately 13.7% (95% CI, 13.60-13.86) of the US population aged 20 to 35 years was Black (Table 1). In contrast, among graduates of programs leading to the 10 occupations that we reviewed, the proportion of Black individuals ranged from 3.1% for PAs to 14.2% for advanced practice registered nurses (Table 4 and Figure, C), resulting in a mean diversity index of 0.54. Hispanic individuals accounted for 21.3% (95% CI, 21.16-21.45) of the US population aged 20 to 35 years (Table 1); however, the proportion of Hispanic graduates of programs leading to the 10 occupations reviewed ranged from 6.6% for pharmacists to 19.0% for RTs (Table 4 and Figure, D). The mean diversity index was 0.48. Native American individuals accounted for 0.7% (95% CI, 0.68-0.73) of the US population aged 20 to 35 years (Table 1), whereas the proportion of Native American graduates among the 10 occupations that we reviewed ranged from 0.2% for OTs to 0.7% for RTs (Table 4), resulting in a mean diversity index of 0.57.

**Table 4.** Graduate Pipeline for Health Diagnosing and Treating Practitioners Based on 2019 Data From the Integrated Postsecondary Education Data System

Occupation	Race/ethnicity, %			
	White	Black	AIAN	Hispanic
Advanced practice registered nurse	63.7	14.2	0.5	11.4
Dentist	56.5	4.9	0.3	9.8
Pharmacist	53.3	9.5	0.4	6.6
Physician	59.5	5.8	0.4	8.5
Physician assistant	77.2	3.1	0.3	7.8
Occupational therapist	78.2	4.2	0.2	7.7
Physical therapist	76.1	3.2	0.3	7.1
Respiratory therapist	54.3	13.3	0.7	19.0
Speech-language pathologist	79.1	3.9	0.4	10.6
Registered nurse	65.2	11.3	0.6	13.6

Abbreviation: AIAN, American Indian or Alaska Native.

The diversity index for the graduate pipeline was lower than 0.50 for 6 of 10 occupations for both Black (dentist, physician, PA, OT, PT, and speech-language pathologist) and Hispanic (dentist, pharmacist, physician, PA, OT, and PT) graduates and for 4 of 10 occupations for Native American graduates (dentist, PA, OT, and PT) (Table 3). The diversity index for recent Black graduates was lower compared with the index of the current workforce in 5 of 10 occupations (PA: 0.23 vs 0.37; OT: 0.31 vs 0.50; PT: 0.23 vs 0.28; speech-language pathologist: 0.28 vs 0.39; registered nurse: 0.82 vs 0.94) (Table 3). For Hispanic graduates, the diversity index decreased compared with the index of the current workforce in 1 occupation (PAs) (Table 4). For Native American graduates, the diversity index decreased compared with the index for the current workforce in 4 occupations (PAs: 0.48 vs 0.94; OTs: 0.24 vs 0.30; RTs: 1.05 vs 1.64; and speech-language pathologists: 0.54 vs 0.96) (Table 3).

## Discussion

Findings of this study showed the extent to which racial/ethnic minority groups are underrepresented in health care professions in the current and future workforce. Although the data show greater diversity among the new graduates who will be entering the health care professions compared with the current health care workforce, the diversity of the educational pipeline remains substantially below the diversity of the general population for almost all health care occupations analyzed in this study. Compared with the representation of Black individuals in the current health care workforce, the representation of recent Black graduates was lower for 5 of the 10 occupations included. Some of the underrepresentation may reflect increases in the educational levels required for entry into some of the professional programs. For example, as of 2020, PA programs must now confer graduate-level degrees to maintain accreditation, and the entry degree for a PT is a doctorate.^29,30^ Although Black PAs and PTs are underrepresented in the workforce, they are even more underrepresented among new graduates, a finding that may be associated with the higher degree requirement.

A lack of diversity in the workforce may reflect a variety of factors, such as low-quality secondary education, limited financial support, lack of mentorship and role models, and unreceptive educational environments. Creating transparency in the data on race/ethnicity may help to increase efforts to address these factors and increase health care workforce diversity. Moreover, the present study provides a comprehensive analysis of the diversity of the health care workforce, which is necessary to improve equity across the health care professions.

Annual reporting on the representation of Black, Hispanic, and Native American people in the health care professions may foster accountability and motivate actions to increase diversity in the health care workforce. Key stakeholders include educational institutions, national associations representing those educational institutions, and state policy makers. All 3 stakeholders can undertake a range of actions to increase the diversity of the health care workforce. We found that nearly all of the diagnosing and treating occupations are below parity, but the parity of some occupations is well below that of others (eg, PAs), even though the years of education required for entry into the profession is less than for other occupations with a better track record on diversity, such as medicine. With regard to the future workforce, for 5 of the 10 occupations included in this cross-sectional study, the low representation of Black graduates in the educational pipeline suggests that the future health care workforce may be less diverse than the existing one. As an industry that self-regulates (ie, sets the requirements for accreditation of programs, including the length of education and curriculum), this outcome is a clear call for introspection and review among leaders of these professions to understand the causes of the lack of racial/ethnic diversity and the steps that can be taken to address these causes and improve health care workforce equity.

We believe that data on racial/ethnic diversity should also inform federal policy makers and serve as the foundation in the design of programs that support increasing diversity. In addition, building and maintaining a comprehensive database on diversity in the health care professions may also facilitate research into factors contributing to increased workforce diversity as well as the association between these factors and health outcomes. To advance this area of inquiry and promote accountability, The George Washington University Fitzhugh Mullan Institute for Health Workforce Equity recently established the Health Workforce Diversity Tracker.^31^ Annual and biennial reports will be generated to track progress by profession, state, and institution to encourage efforts to have an educational pipeline that more closely resembles the population.

### Limitations

This study has several limitations. First, although the ACS is a nationally representative sample survey that provides population and occupation estimates, the estimates by race/ethnicity reflect small samples and may be subject to sampling error. The SEs for the population samples used in this study were all less than 1, suggesting that the sample was representative of the population. Unlike the ACS, data from the IPEDS included all awards and degrees conferred between July 1, 2018, and June 30, 2019, in the US. Although 2019 data on completed degree coursework appeared to include nearly all graduates, there was a small chance that administrative errors from reporting by thousands of schools submitting data on graduates in hundreds of categories may affect findings from these data.

Estimates reported herein rely on the most recent releases of ACS and IPEDS data (2019 one-year estimates). Restricting the analysis to 1 year of ACS and IPEDS data may not account for minor year-to-year variations in the data. In preparing this analysis, estimates from the last 5 years of ACS and IPEDS data were compared and found to be relatively stable over time, particularly in the last 3 years. It is also worth noting that this study excluded individuals who self-reported race as mixed, other, or multiple races, a population that has been steadily growing over time. Greater attention to this growing population is needed, and future research needs to ensure that categorization of this group is comparable across ACS and IPEDs data.

A key component of the present analysis is the use of the diversity index, which was designed to adjust for differences in the diversity of the population by race/ethnicity or changes in the diversity of the general population over time. This tool also may be helpful for comparisons of the extent of diversity across states that have very different racial/ethnic makeups and changes over time as the population composition changes. A limitation of the index is that it may mask a wide range of performance within a profession by institution and in different regions of the country.

Another limitation is the assumption that all graduates go on to become practitioners and that the diversity of the education pipeline will reflect the diversity of new practitioners. It is possible that some graduates do not go on to practice, and this factor could vary by race/ethnicity.

## Conclusions

This cross-sectional study comparing the racial/ethnic diversity among 10 health care professions found that Black, Hispanic, and Native American people were underrepresented compared with their representation in the general population. Although there has been some improvement in diversity among graduates of health care professional programs compared with the current workforce, this study’s findings suggest a need for additional policies to support a health care workforce that is representative of the diversity of the current population. Measuring, tracking, and regularly reporting on the extent of representation of Black, Hispanic, and Native American health workers can encourage health care professional organizations, states, and individual institutions to make greater efforts to increase representation.
